# Acupuncture Ameliorates Depression-Like Behaviors Through Modulating the Neuroinflammation Mediated by TLR4 Signaling Pathway in Rats Exposed to Chronic Restraint Stress

**DOI:** 10.1007/s12035-023-03737-6

**Published:** 2023-11-02

**Authors:** Huili Jiang, Xianqi Long, Yu Wang, Xuhui Zhang, Lu Chen, Xinjing Yang, Bingcong Zhao, Ye Zhang, Yemao Chai, Tuya Bao

**Affiliations:** 1 School of Acupuncture-Moxibustion and Tuina, Beijing University of Chinese Medicine, No. 11, Bei San Huan Dong Lu, Chaoyang District, Beijing, 100029 China; 2 Research Center of Mental and Neurological Disorders, School of Acupuncture-Moxibustion and Tuina, Beijing University of Chinese Medicine, No. 11, Bei San Huan Dong Lu, Chaoyang District, Beijing, 100029 China; 3 Department of Medicine, Qiannan Medical College for Nationalities, Duyun, China; 4 Institute of Acupuncture and Moxibustion, China Academy of Chinese Medical Sciences, Beijing, China; 5 Beijing HaiDian Hospital, Beijing, China; 6 Department of Traditional Chinese Medicine, South China Hospital of Shenzhen University, Shenzhen, China; 7 Beijing Key Laboratory of Acupuncture Neuromodulation, Acupuncture and Moxibustion Department, Beijing Hospital of Traditional Chinese Medicine, Capital Medical University, Beijing, China; 8 Dongfang Hospital, Beijing University of Chinese Medicine, Beijing, China

**Keywords:** Acupuncture, Depression, Neuroinflammation, TLR4 signaling pathway, Stress, Microglia

## Abstract

Recently, emerging evidence has identified that stress-induced activation of neuroinflammation is considered to be one of the most prevalently precipitating factors in the pathogenesis of depression. Data from clinical trials and experimental findings has verified the efficacy and safety of acupuncture in the prevention and treatment of depression. However, the mechanism of the preventive effect of acupuncture for depression has not been fully elucidated. The current study aimed to investigate the preventive effect and mechanism of acupuncture through modulating the neuroinflammation mediated by toll-like receptor 4 (TLR4) signaling pathway in rats exposed to chronic restraint stress (CRS). All rats were subjected to CRS for 21 days, with the exception of rats in control group. One hour before CRS, rats in acupuncture group were exposed to acupuncture at Baihui (GV20) and Yintang (GV29). The depression-like behaviors were evaluated by body weight assessment and sucrose preference test at 0, 7, 14, and 21 days. The expression of activated microglia in hippocampus was detected by immunofluorescence. The expression of key proteins on TLR4 signaling pathway of TLR4, MyD88, TRAF6, NF-κB p65, TNF-α, and mRNA of TLR4 in the hippocampus was detected by western blot and real-time quantitative polymerase chain reaction to investigate the effect of acupuncture on stress-induced activation of neuroinflammation. The present study provided evidence that acupuncture exerted potential preventive effect that might be mediated in part by suppressing the neuroinflammation induced by TLR4 signaling pathway, which may be a promising treatment target to improve current treatments for depression.

**Supplementary Information:**

The online version contains supplementary material available at 10.1007/s12035-023-03737-6.

## Introduction

Depression, one of the most prevalent psychiatric disorders, is characterized by low mood, loss of interest, guilty feelings or low self-esteem, sleep and appetite disorders, anxiety, and anhedonia [1], even exposed to increased risk of suicide [2]. Data from the epidemiological investigation have indicated that depression is considered to be the current primary cause of disability worldwide [3]. The World Health Organization (WHO) predicts that depression could be the leading cause of the greatest global burden by 2030 [4]. Before 2020, mental disorders have been reported to be the leading causes of the global health-related burden, with depressive and anxiety disorders being leading contributors to this burden. Notably, the emergence of the coronavirus disease 2019 (COVID-19) pandemic has created an environment where many determinants of poor mental health are exacerbated. It has been reported that throughout 2020, the pandemic led to a 27.6% increase in cases of major depressive disorders and 25.6% increase in cases of anxiety disorders globally [5–7]. Despite these increasing studies and significant economic costs, the pathogenesis and the molecular mechanism underlying depression are still not fully understood [8]. Presently, various different pharmacological approaches have been widely used in the treatment of depression. Selective serotonin reuptake inhibitors (SSRIs), such as paroxetine, fluoxetine, or escitalopram, are the clinically first-line antidepressants. However, findings from the clinical investigation have indicated that the response rate (defined as at least a 50% improvement from baseline) to SSRIs is only about 60%, which even is accompanied with confirmed side effects such as nausea, vomiting, or sexual function recession [9, 10]. In addition, the experiment observation has reported that fluoxetine treatment could induce anxiogenic-like responses in the animal model of depression [11]. Accordingly, it is sensible to explore effective strategies to improve current treatment and prevention for depression.

Accumulating evidence from clinical investigation and experimental study has identified the involvement of the stress-induced activation of inflammation, including neuroinflammation and systemic inflammation in the development and pathogenesis of depression [12–14]. It has been identified that depressive symptoms can be induced in humans with administration of low-dose lipopolysaccharide (LPS), which could activate the innate immune system and trigger the release of inflammatory cytokines [15]. The increased levels of biomarkers of inflammation such as inflammatory cytokines and acute-phase proteins have been found to be reliably elevated in a significant proportion of patients suffered from major depressive disorder (MDD) [16]. The experimental findings have also indicated that the CD-1 mice subjected to the administration of interleukin-1beta (IL-1β) and LPS contributed to depression-like effects in the tail suspension test (TST) and the forced swim test (FST) [17]. The previous studies have also reported that the obviously increased peripheral and central proinflammatory cytokines, including hypothalamus, hippocampus, pituitary, and spleen, were induced by exposure to stress in animal model [18], suggesting the specific role of stress-induced inflammatory reaction in the pathogenesis of depression. Our recent studies have also demonstrated that exposure to chronic restraint stress (CRS) triggered the significant activation of microglia and neuroinflammation induced by high mobility group box-1 (HMGB1), and induced depression-like behaviors in rats subjected to CRS [19], which indicated that stress-activated neuroinflammation might be the key pathway linking stress and depression.

Toll-like receptors (TLRs), affiliated to the germline-encoded pattern recognition receptors (PRRs), have been identified to be the important mediators of inflammatory pathways in the pathological process of depression, which plays a major role in the subsequent initiation of immune responses [19, 20]. Previously, twelve members of the TLR family have been identified in the mammals [21]. Among the TLR family, toll-like receptor 4 (TLR4) has been demonstrated to be one potential inflammatory regulator which is considered to be associated with MDD [22]. Clinical investigation have shown that increased expression of TLR-4 mRNA and protein, as well as NF-κβ are found in newly diagnosed patients with MDD, suggesting the involvement of the stress-induced neuroinflammation mediated by the TLR4 pathway [23, 24]. And clinical improvement of depressive symptoms during psychotherapy has been identified to be associated with decreased expression of pro-inflammatory markers mediated by TLR4 [23]. Another clinical study has also reported that TLR mRNA levels were differentially expressed in MDD patient and TLR4 was found to be an independent risk factor associated with the severity of MDD [25]. Findings from the previous studies have reported that the observed abnormalities of proinflammatory cytokines in the brain of suicide victims may be related to an abnormality of TLR4 over-expression [26]. Moreover, results in the animal study have confirmed that LPS from bacterial translocation contributed to the TLR 4 activation in rats subjected to chronic mild stress (CMS), which leads to release of inflammatory mediators offers a potential preventive approach for the treatment of depression [27]. Another study has also demonstrated that the inflammatory process driven by TLR4/myeloid differentiation factor88 (MyD88)/nuclear factor kappa-B (NF-κB) signaling pathway plays a critical role in the chronic unpredictable mild stress (CUMS)-induced depression-like behavior, including the significant decrease in preference for a sucrose solution or in total moving distance [28]. And the antidepressants obviously alleviated CUMS-induced depression-like behavior and exerted considerable neuroprotective effects by regulating the microglial state transition by inhibiting the TLR4/MyD88/NF-κB signaling pathway [28]. Our previous studies about genome-wide transcriptome analysis of hippocampus have also indicated that TLR signaling pathway was involved in the pathogenisis of stress-induced depressive disorder [29].

Currently, data from clinical trials and experimental funding has verified the effectiveness and safety of acupuncture for depression [30–33]. Our preliminary findings have confirmed that acupuncture could regulate the stress-induced activation of neuroinflammation and exert antidepressant effect [19, 34, 35], which has also been supported by the data from clinical investigations. However, the mechanism of the preventive effect of acupuncture for depression through modulating the stress-induced activation of neuroinflammation mediated by TLR4 signaling pathway has not been fully elucidated. Accordingly, here the rat model in the present study was established by exposure to CRS to simulate depression based on the preliminary research of our team. The expression of the key hippocampal proteins and mRNAs on TLR4 signaling pathway was detected. We aimed to elucidate the mechanisms underlying the preventive effect of acupuncture through regulating the upper triggers of the stress-induced activation of neuroinflammation mediated by TLR4 signaling pathway, which might shed new light on conceptual frameworks of prospects for new strategies for depression.

## Materials and Methods

### Animals and Experimental Grouping

**Fig. 1:**
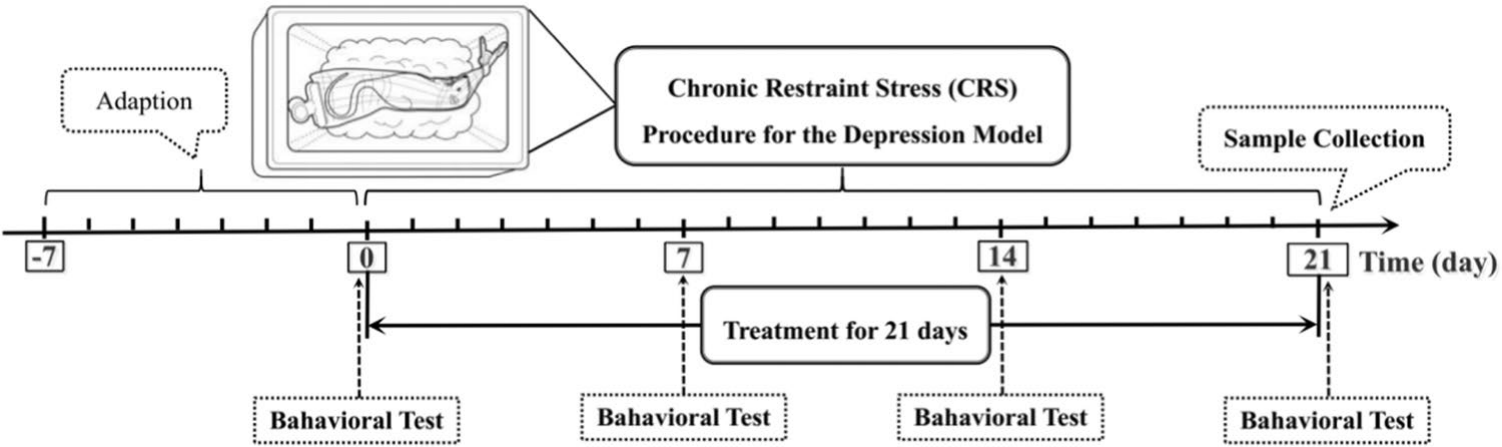
The experimental flow chart

Adult male Sprague-Dawley rats, weighting 180±10 g, were obtained from the Weitong Lihua Experimental Animal Center (Beijing, China). Animal care and the experiments were carried out in keeping with the agreement of Animal Ethics Committee, Beijing University of Chinese Medicine, China (Permission number: BUCM-4-2020102801-4048). All rats were housed and adapted for 7 days in a controlled environment of 24–26 °C and 50%±10% humidity, with a 12-h light/dark cycle. After 7 days of adaption, the rats with the same baseline of behavioral assessment were enrolled in the present study. A total of 44 rats were included in the present study after behavioral assessment to guarantee the consistency of baseline characteristics. Rats were randomly divided into control group, model group, escitalopram group, and acupuncture group, with 11 rats in each group (Fig. 1).

### Chronic Restraint Stress Procedure for the Depression Model

Except rats in control group, all rats were subjected to social isolation and CRS procedures for 21 days continuously in the present study. Studies have confirmed that CRS procedures can well simulate the pathological process of depression [36]. Accordingly, the establishment of the animal model of depression in the present study was conducted to be the validation of CRS referring to the previous study [19, 29, 36]. Rats were restrained in a cylinder-shaped wire net (20.5-cm long and 6.5-cm in diameter), fixing both ends with a 64-mm-long butterfly clip from 10 a.m. to 4 p.m. The wire net was soft enough to prevent from body impairment of rats. During CRS procedures, all rats were subjected to food and water deprivation. After CRS procedures, all rats were put back into the cage and they had free access to food and water.

### Acupuncture and Escitalopram Intervention

Rats in acupuncture group were acupunctured at Baihui (GV 20) and Yintang (GV 29) compatibly with our previous described procedures [19, 29]. Acupuncture was conducted 1 h before CRS procedures, 20 min per session, and 1 session daily for 21 days. Following disinfection of the acupoint sites with 75% alcohol, the acupuncture needles (0.3 mm in diameter and 25-mm long; Suzhou Acupuncture & Moxibustion Appliance Co., Ltd., Jiangsu, China) were inserted transversely (keeping the angle between the needle and the skin surface at 15 angle) into Baihui (GV 20) and Yintang (GV 29) to a depth of 5 mm. Acupoints coordinates (GV 20, located at the bregma, or on the junction of coronal suture and sagittal suture; GV 29, midway between the medial ends of the two eyebrows) were under the guidance as described previously [24, 34]. When rats in acupuncture group were exposed to acupuncture stimulation, rats were placed in separated room and under the conditions of free activities. Rats in escitalopram group were administered with the suspension of escitalopram and saline administration (30 mg/100 ml) by gavage (3 mg/kg day) 1 h before CRS procedure, once daily for 21 days. The escitalopram oxalate tablets were obtained from the Xian Janssen Pharmaceutical Ltd. (Xi’aan, China).

### CRS-Induced Depression-Like Behavior Test

All behavioral tests were conducted under relatively quiet and dark circumstances. Body weight assessment and sucrose preference test (SPT) were performed at 0 day pre-intervention and at 7, 14, and 21 days post-intervention. The changes of body weight were detected to evaluate the states of food preference and nutrition status. SPT was investigated to evaluate the CRS-induced anhedonia. Procedures of SPT were performed according to the previous studies [19, 29, 37]. Sucrose preference index was estimated as follows. Sucrose preference index (%) = [sucrose intake/ (sucrose intake + pure water intake)] × 100%. Anhedonia was expressed by reduced sucrose preference.

### Tissue Collection and Processing Procedures

No rats died during the experiment. At the end of the experimental procedures, rats were anesthetized with an injection of 10% chloral hydrate (0.35 ml/100 g, i.p.) for sample collection. The blood samples of 8 rats in each group were randomly drawn from the retro orbital plexus and centrifuged at 3000 rpm for 15 min to separate serum, which were kept at –80 °C for the detection of the contents of the cytokines in serum by enzyme-linked immunosorbent assay (ELISA). Then the hippocampus were harvested on the ice immediately after decapitation, washed with cold phosphate buffered saline (PBS), dried, and weighed. Then the hippocampus was immediately kept at –80 °C for the detection of the key proteins and mRNAs of TLR4 signaling pathway. Samples with obvious protein degradation were excluded after BCA assay. Then 5 samples of the left hippocampal tissue were homogenized for western blot (WB) analysis and 6 right hippocampal tissue for quantitative real-time PCR analysis. Meanwhile, another 3 rats in each group were anesthetized with 10% chloral hydrate and perfused transcardially with 0.9% saline followed by 4% paraformaldehyde solution. The intact brain was removed after perfusion and fixed in 4% paraformaldehyde solution for the H&E staining and immunofluorescence staining.

### H&E Staining

The pre-processed intact brain was embedded in OCT (Tissue-Tek) and cut at a 5-μm thickness after full fixation by 4% paraformaldehyde solution. These slices were stained with hematoxylin/eosin (H&E) staining and observed under an optical microscope (Carl Zeiss Microscopy, Germany) to evaluate histopathological changes of the hippocampus.

### Immunofluorescence Staining

The intact brain tissue was fixed with 4% paraformaldehyde for the immunofluorescence staining. The brain samples were embedded in OCT and serial sectioned into 10-μm thickness along the coronal plane with cryostat (CRYOSTAR NX50, Thermo, USA). Then the hippocampus was mounted on glass slides for the next step. After all the sections in each group were repaired with EDTA antigen repair buffer, non-specific binding was blocked with 4% donkey serum for 60 min at room temperature. The slides were washed with 1 × TBS containing 0.1% Tween-20 and then exposed overnight with the specific primary antibody mixtures: IBA1 Rabbit Polyclonal antibody (1:500, Proteintech, 10904-1-AP, USA) at 4 °C. Then the detection of primary antibodies was performed with secondary antibody (Goat Anti-Rabbit IgG H&L (HRP) (1:1000, Abcam, ab6721, USA)) for 2 h in the darkness. The sections were then washed five times with PBS. And the nucleus was restained with DAPI staining solution (1:1000, Abcam, ab228549, USA) and then incubated at room temperature for 5 min. The slides were washed with PBS for 3 times, air dried for 30 min at room temperature. The anti-fluorescent extractant was added. Immunofluorescence sections were observed. Ultimately, the immunofluorescence results were observed and scanned with the fluorescence microscope (Nikon Eclipse Ti-SR, Japan) and immunofluorescence scanning instrument (Pannoramic MIDI, 3D Histech). Hippocampal fluorescent images were captured by the Case Viewer soft.

### Western Blot Analysis

The expression of key proteins of TLR4 signaling pathway was detected by western blot analysis. Five samples of the left hippocampal tissue were homogenized in lysis buffer with protease inhibitors and ready for WB. The primary antibodies were added and then incubated at 4 overnight with the following antibodies: anti-TLR4 (1:1000, ab32127, Abcam), anti-MyD88 (1:1000, ab22048, Abcam), anti-TRAF6 (1:1000, ab2064, Abcam), anti-NF-κB-65 (1:1000, ab40675, Abcam), anti-IL-1 beta (1:1000, ab16502, Abcam), and β-actin (1:10000, TA-09, ZSGB-Bio Co., Ltd., China). After washing with TBS containing 0.1% Tween-20, blots were incubated in horseradish peroxidase-conjugated secondary antibody (1:1000, 111-035-003, Jackson) for 40 min at room temperature. The membrane was visualized using a chemiluminescence kit (WBKLS0500, Millipore) according to the manufacturer’s instruction. The signal intensities from the immunoblots were analyzed by densitometry. The analyses were normalized to β-actin.

### Quantitative Real-time PCR Analysis

The expression of the mRNA of TLR4, MyD88, and NF-κB p65 in the hippocampus was detected by quantitative real-time PCR analysis in accordance with basic procedures. The total RNA was extracted from 50-mg hippocampus tissues using Trizol reagent (Invitrogen, USA). The cDNA was synthesized using Eastep RT Master Mix. Amplification conditions was 95 °C 10 min for polymerase activation/denaturation, and (95 °C for 10 s, 59 °C for 60 s)× 45 cycles at cycling stage. SYBR FAST qPCR Kit Master Mix(2×) Universal (KAPA Biosystems) and ABI PRISM 7500 Sequence Detection System (ABI7500, USA) were used for qPCR. GAPDH was selected to be the reference gene. Primers were designed using the Primer-Blast program from NCBI. The primer sets used were as the following. TLR4, forward: F3′-TCCACAAGAGCCGGAAAGTT-5′, reverse: R3′-CCAGAGCGGCTACTCAGAAA-5′; MyD88, forward: F3′-CTCGCAGTTTGTTGGATGCC-5′, reverse: R3′-GCGACTTCAGCTCCTTCAGT-5′; NF-κB p65, forward: F3′-CAATCACGATCGTCACCGGA-5′, reverse: R3′-TCTGCCCAGAAGGAAACACC-5′; GAPDH, forward: F3′-CTTTGGCATTGTGGAAGGGC-5′, reverse: R3′-CAGGGATGATGTTCTGGGCA-5′, with the predicted sizes 115 bp, 111 bp, 245 bp, and 125 bp, respectively. The 2^−∆∆Ct^ method [∆CT = (Ct_target_ − Ct_GAPDH_)] was then used to convert 1 CT values to mRNA fold changes relative to the control group. The mRNA levels of the targets were normalized with glyceraldehyde-3-phosphatedehydrogenase (GAPDH) mRNA level to exclude effects of varying RNA amounts.

### ELISA Assay

The content of serum IL-1β and IL-10 was detected by enzyme-linked immunosorbent assay (ELISA) method according to the manufacturer’s protocol from the ELISA assay kit (Rat IL-1β ELISA kit, E02I0010, BlueGene, China; Rat IL-10 ELISA kit, E02I0023, BlueGene, China).

### Data Analysis

All data were statistically analyzed by SPSS 22.0 software package (IBM, Armonk, NY, USA). Data were presented as the mean ± standard error of the mean (S.E.M.). Within the data of body weight, sucrose preference rate at different time points, two-way analysis of variance (ANOVA) was selected for data consistent with normal distribution and homogeneous variance, and least-significant difference (LSD) post hoc test was followed during pairwise comparisons between groups. Additionally, one-way ANOVA were used for the other data. Differences between individual means were tested for significance using Fisher’s least significant difference (LSD) procedure. All statistical results were statistically significant at *p* < 0.05.

## Results

### Acupuncture Alleviated the CRS-Induced Depression-Like Symptoms

**Fig. 2:**
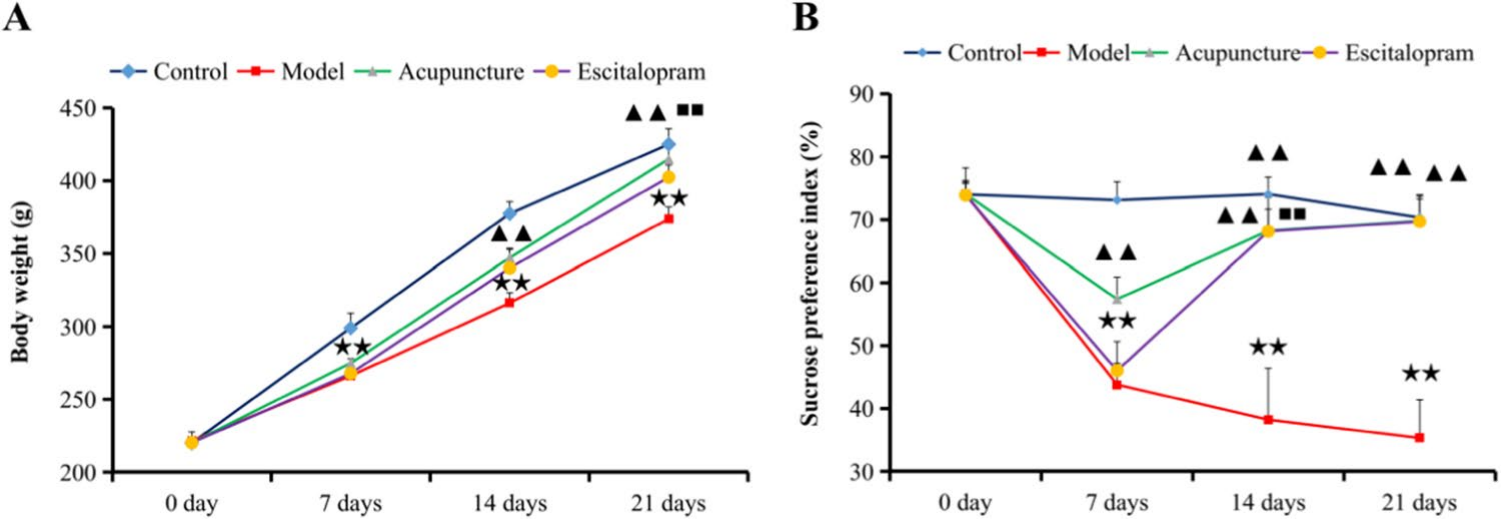
Acupuncture alleviated the CRS-induced depression-like symptoms. **A** Effect of acupuncture on the changes of body weight of CRS rats. **B** Effect of acupuncture on the changes of sucrose preference index of CRS rats. ^★★^*P*˂0.01 compared with the control group; ^▲▲^*P*˂0.01 compared with the model group; ^■■^*P*˂0.01 compared with the escitalopram group

In the present study, body weight and sucrose preference test (SPT) were used to detect the changes of appetite and anhedonia respectively, and to evaluate the effectiveness of acupuncture on the depression-like symptoms in rats exposed to CRS. As shown in Table 1 and Fig. 2A, before the CRS procedure, there was no significance in the body weight of each group. After the CRS procedure, the body weight of each group at different time points was significantly different (*F*=109.873, *P*=0.000). The body weight of rats in the model group was significantly lower compared to that of the rats from the control group after 7 days of CRS procedures, even lower than that in the control group significantly until at day 21 (all *P* < 0.01). Notably, acupuncture intervention significantly increased the body weight of the rats compared to that of the rats from model group at days 7, 14, and 21 (all *P* < 0.01).

**Table 1:** Effect of acupuncture on the changes of body weight of CRS rats (*n*=11; g)

Group	0 day	7 days	14 days	21 days
Control	220.091±0.782	298.991±10.198	377.391±8.202	425.027±10.589
Model	221.582±6.143	266.109±3.572^★★^	316.173±7.031^★★^	373.682±8.284^★★^
Escitalopram	220.245±1.951	267.773±4.564	340.100±13.539^▲▲^	402.364±8.378^▲▲^
Acupuncture	220.373±2.105	274.864±3.174^▲▲;■■^	347.164±5.721^▲▲^	414.827±8.544^▲▲;■■^

Notes: Data were expressed as mean ± S.E. (*n* = 11; g). *F*=14.750, *P*=0.000. ^★★^*P*˂0.01 compared with the control group; ^▲▲^*P*˂0.01 compared with the model group; ^■■^*P*˂0.01 compared with the escitalopram group

As shown in Table 2 and Fig. 2B, before the CRS procedure, there was no significance in the sucrose preference index of each group. However, post-exposure sucrose preference index was significantly different during each group (*F*=221.345, *P*=0.000). Compared with the control group, the sucrose preference index of rats in the model group was significantly lower after 7 days of CRS procedures, even lower than that in the control group significantly until at day 21 (all *P* < 0.01), which indicated the CRS-induced anhedonia and depressive-like behavior. Compared with the model group, acupuncture and escitalopram intervention reversed the CRS-induced anhedonia. And acupuncture intervention significantly increased the sucrose preference index compared to that in model group at days 7, 14, and 21 (all *P* < 0.01), suggesting that acupuncture can significantly exert preventive effects in rats subjected to CRS procedures (Table 2).

**Table 2:** Effect of acupuncture on the changes of sucrose preference index of CRS rats (*n*=11; %)

Group	0 day	7 days	14 days	21 days
Control	74.012±1.744	73.117±2.928	74.063±2.727	70.341±3.405
Model	74.056±4.190	43.806±3.390^★★^	38.241±8.142^★★^	35.320±6.084^★★^
Escitalopram	73.897±2.352	46.034±4.612	68.137±3.534^▲▲^	69.699±3.573^▲▲^
Acupuncture	74.096±1.876	57.399±3.488^▲▲;■■^	68.232±5.299^▲▲^	69.801±4.137^▲▲^

Notes: Data were expressed as mean ± S.E. (*n* = 11; %). *F*=221.345, *P*=0.000. ^★★^*P*˂0.01 compared with the control group; ^▲▲^*P*˂0.01 compared with the model group; ^■■^*P*˂0.01 compared with the escitalopram group

### Acupuncture Attenuated the CRS-Induced Pathological Changes in the Hippocampus

**Fig. 3:**
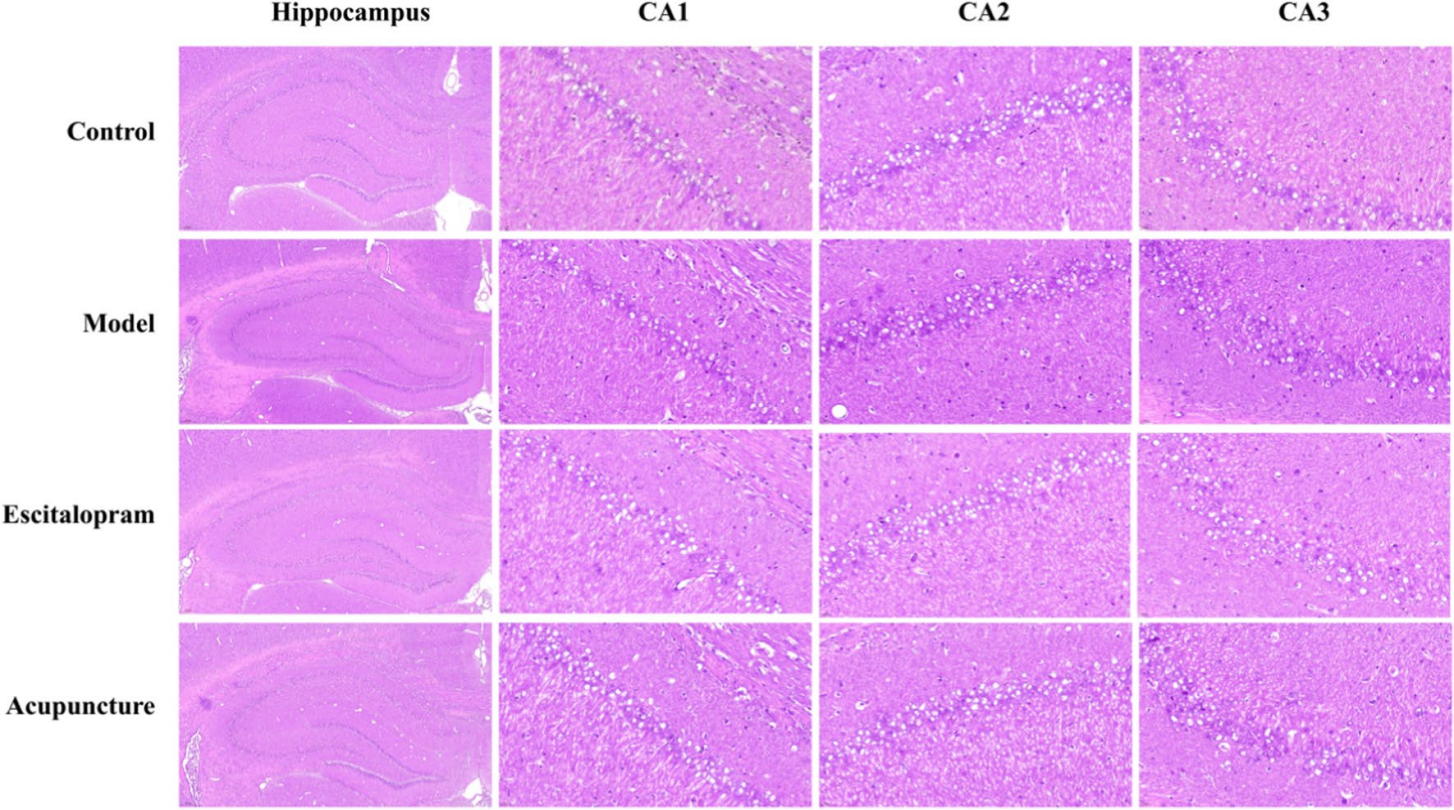
Effect of acupuncture on the pathological change in the hippocampus of CRS rats (× 400). Scale bar, 20 μm

The severity of pathological changes was detected by morphological observations (Fig. 3). Results of the hematoxylin/eosin staining showed that the hippocampus neurons of CA1/2/3 region in the control group were orderly arranged with normal structure, clear nuclei, and abundant cytoplasm. After the CRS procedure, increasing swollen neurons with loosen structure, karyopyknosis, and forming some vacuolar structures could be obviously observed in the model group, especially in CA3 region. Compared with model group, the pathological changes of hippocampus neurons were significantly alleviated in both acupuncture group and escitalopram group.

### Acupuncture Downregulated the Expression of IBA-1 in the Hippocampus of CRS Rats

**Fig. 4:**
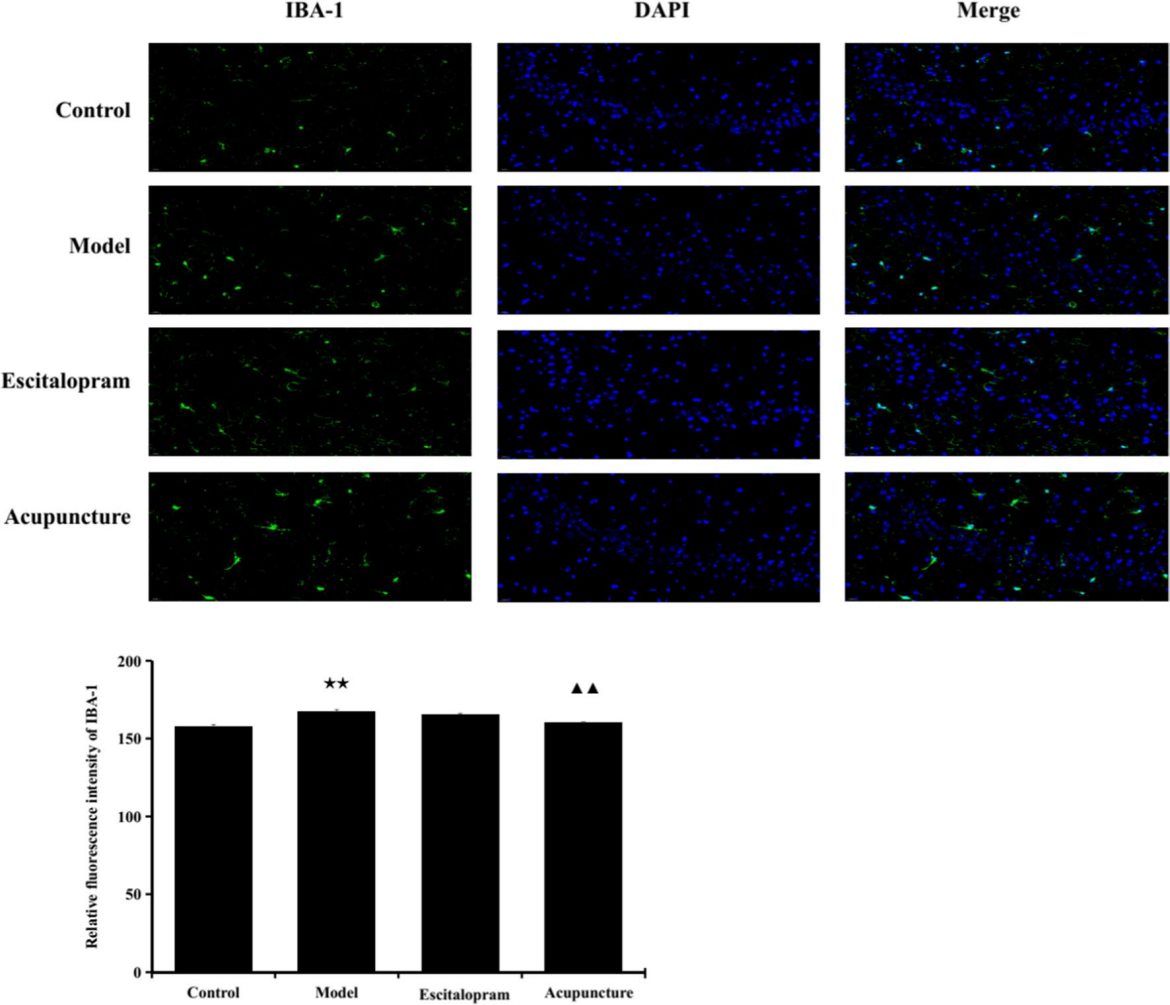
Effect of acupuncture on the expression of IBA-1 in the hippocampus of CRS rats. **A** Immunofluorescence staining of hippocampal CA3 region in different groups (×40). IBA-1, green; DAPI, blue; scale bar, 20 μm; **B** Relative fluorescence intensity of IBA-1. Data was expressed as means ± S.E. (*n* = 5). ^★★^*P*˂0.01 compared with the control group; ^▲▲^*P*˂0.01 compared with the model group. One-way ANOVA followed by LSD’s post hoc test

As shown in Fig. 4 and Table 3, after exposure to 21 days of CRS procedures, there was a significant difference in the expression of IBA-1 in the hippocampus CA3 region among groups (*F* = 19.169, *P* = 0.000). The expression of IBA-1 was significantly higher than that of the control group (*P*< 0.01), suggesting the involvement of stress-induced microglia activation in the pathogenesis of depression, while acupuncture intervention reversed these effects and decreased the expression of IBA-1 in the hippocampus CA3 region compared to model group (*P*< 0.01).

**Table 3:** Effect of acupuncture on the expression of IBA-1 in the hippocampus of CRS rats

Group	The relative fluorescene intensity of IBA-1
Control	157.664±1.390
Model	167.330±1.325^★★^
Escitalopram	165.702±0.551
Acupuncture	160.151±0.586^▲▲^

Notes: Data was expressed as means ± S.E., one-way ANOVA followed by LSD’s post hoc test (*n* = 5); *F*=19.169, *P˂0*.01. ^★★^*P*˂0.01 compared with the control group; ^▲▲^*P*˂0.01 compared with the model group

### Acupuncture Ameliorated the CRS-Induced Neuroinflammation Mediated by TLR4 Signaling Pathway in the Hippocampus of CRS Rats

**Fig 5:**
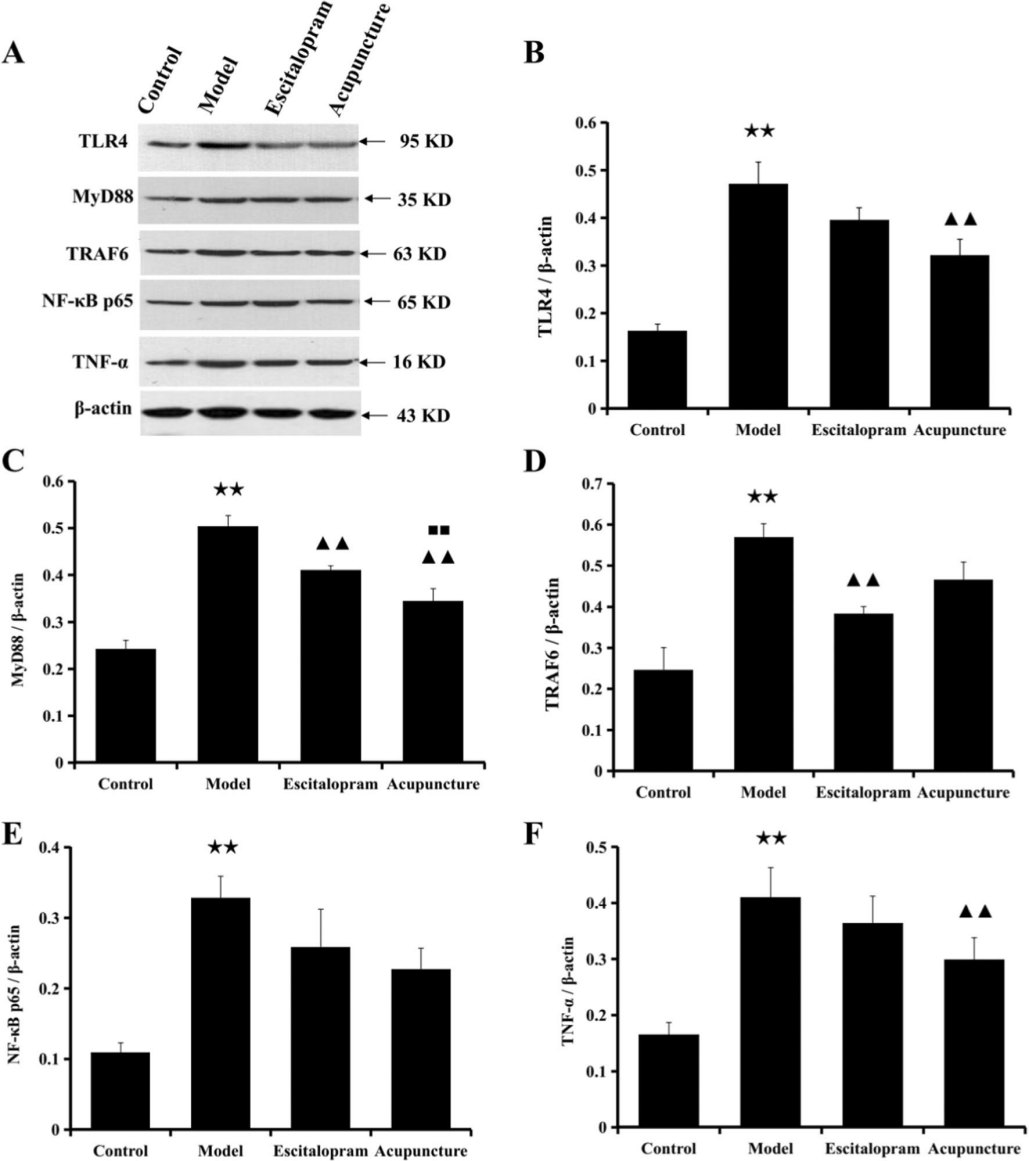
Effect of acupuncture on the expression of the key protein levels of TLR4 signaling pathway in the hippocampus of CRS rats. **A** The protein band diagram of TLR4, MyD88, TRAF6, NF-κB p65, and TNF-α, and **B**–**F** the bar diagram of TLR4, MyD88, TRAF6, NF-κB p65, and TNF-α. Data were expressed as mean ± S.E. (*n* = 5). ^★★^*P*˂0.01 compared with the control group; ^▲▲^*P*˂0.01 compared with the model group; ^■■^*P*˂0.01 compared with the escitalopram group. One-way ANOVA followed by LSD’s post hoc test. *TLR4*, toll-like receptor 4; *MyD88*, myeloid differentiation primary response gene 88; *TRAF6*, TNF receptor-associated factor 6; *NF-κB p65*, nuclear factor kappa B p65; *TNF-α*, tumor necrosis factor α

As illustrated in Table 4 and Fig. 5, the expression of key proteins on TLR4 signaling pathway of TLR4, MyD88, TRAF6, NF-κB p65, and TNF-α in the hippocampus was detected by western blot. Compared with the control group, CRS exposure significantly contributed to the increase in the expression of TLR4, MyD88, TRAF6, NF-κB p65, and TNF-α in the hippocampus (TLR4, *F*=16.748, *P*=0.000; MyD88, *F*=29.210, *P*=0.000; TRAF6, *F*=12.011, *P*=0.000; NF-κB p65, *F*=6.976, TNF-α, *P*=0.003; *F*=6.394, *P*=0.005; all *P* < 0.01), indicating the involvement of the stress-induced activation of neuroinflammation in the pathogenesis of depression. Of note, acupuncture reversed the CRS-induced increase in the expression of TLR4, MyD88, and TNF-α in the hippocampus (all *P* < 0.01), which suggested that the stress-induced activation of neuroinflammation was significantly alleviated by acupuncture compared to the model group.

**Table 4:** Effect of acupuncture on the expression of the key protein levels of TLR4 signaling pathway in the hippocampus of CRS rats

Group	TLR4/β-actin	MyD88/β-actin	TRAF6/β-actin	NF-κB p65/β-actin	TNF-α/β-actin
Control	0.163±0.014	0.242±0.019	0.247±0.054	0.109±0.014	0.165±0.022
Model	0.471±0.046^★★^	0.504±0.023^★★^	0.569±0.033^★★^	0.328±0.031^★★^	0.410±0.053^★★^
Escitalopram	0.395±0.026	0.410±0.010^▲▲^	0.384±0.017^▲▲^	0.259±0.053	0.364±0.048
Acupuncture	0.321±0.034^▲▲^	0.345±0.026^▲▲,■■^	0.466±0.043	0.227±0.030	0.299±0.039^▲▲^

Notes: Data were expressed as mean ± S.E. (*n* = 5). TLR4, *F*=16.748, *P*=0.000; MyD88, *F*=29.210, *P*=0.000; TRAF6, *F*=12.011, *P*=0.000; NF-κB p65, *F*=6.976, *P*=0.003; TNF-α, *F*=6.394, *P*=0.005. ^★★^*P*˂0.01 compared with the control group; ^▲▲^*P*˂0.01 compared with the model group; ^■■^*P*˂0.01 compared with the escitalopram group. *TLR4*, toll-like receptor 4; *MyD88*, myeloid differentiation primary response gene 88; *TRAF6*, TNF receptor-associated factor 6; *NF-κB p65*, nuclear factor kappa B p65; *TNF-α*, tumor necrosis factor α

### Acupuncture Positively Regulated the Expression of TLR4, MyD88, and NF-κB p65 mRNA in the Hippocampus of CRS Rats

**Fig. 6:**
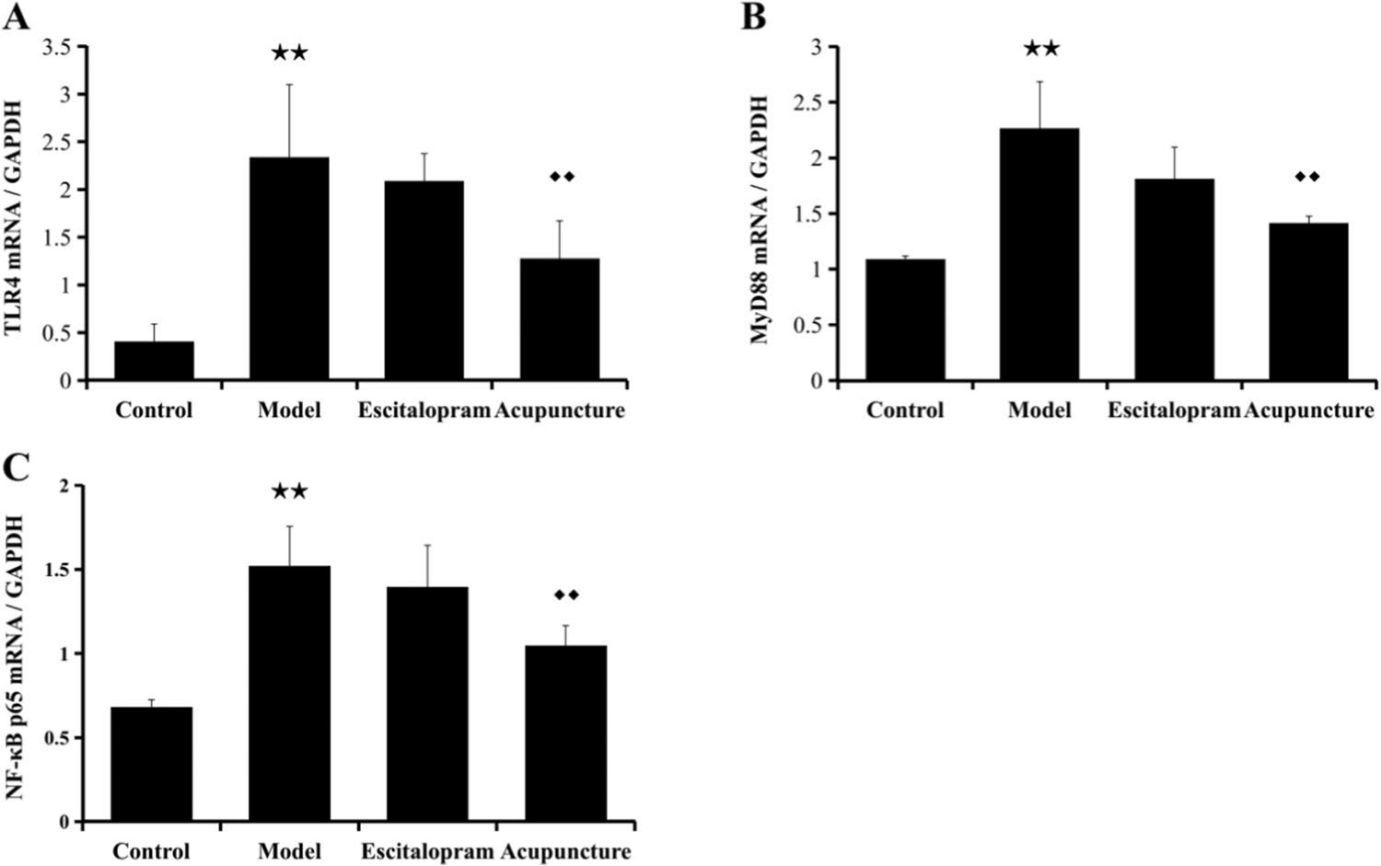
Effect of acupuncture on the expression of TLR4, MyD88, and NF-κB p65 mRNA in the hippocampus of CRS rats. **A**–**C** The bar diagram of TLR4, MyD88, and NF-κB p65 mRNA in the hippocampus. Data are expressed as means ± S.E. (*n* = 6). Data were expressed as mean ± S.E.. ^★★^*P*˂0.01 compared with the control group; ^◆◆^*P*˂0.05 compared with the model group. One-way ANOVA followed by LSD’s post hoc test. *TLR4*, toll-like receptor 4; *MyD88*, myeloid differentiation primary response gene 88; *NF-κB p65*, nuclear factor kappa B p65

As shown in Table 5 and Fig. 6, the expression of TLR4, MyD88, and NF-κB p65 mRNA in the hippocampus was detected by Q-PCR. The changes of the expression of TLR4, MyD88, and NF-κB p65 mRNA in the hippocampus were basically consistent with the key protein levels of TLR4 signaling pathway. After 21 days of CRS exposure, the mRNA expression of TLR4, MyD88, and NF-κB p65 was upregulated (TLR4, *F*=3.505, *P*=0.034; MyD88, *F*=3.867, *P*=0.025; NF-κB p65, *F*=4.190, *P*=0.019) than that of the control group (all *P* < 0.01), which could be downregulated after the acupuncture intervention (all *P* < 0.05).

**Table 5:** Effect of acupuncture on the expression of TLR4, MyD88, and NF-κB p65 mRNA in the hippocampus of CRS rats

Group	TLR4 mRNA/GAPDH	MyD88 mRNA/GAPDH	NF-κB p65 mRNA/GAPDH
Control	0.403±0.189	1.090±0.029	0.683±0.042
Model	2.333±0.767^★★^	2.263±0.423^★★^	1.520±0.236^★★^
Escitalopram	2.083±0.292	1.810±0.287	1.393±0.251
Acupuncture	1.273±0.400^◆◆^	1.410±0.067^◆◆^	1.047±0.119^◆◆^

Notes: Data were expressed as mean ± S.E. (*n* = 6). TLR4, *F*=3.505, *P*=0.034; MyD88, *F*=3.867, *P*=0.025; NF-κB p65, *F*=4.190, *P*=0.019. ^★★^*P*˂0.01, compared with the control group; ^◆◆^*P*˂0.05, compared with the model group. One-way ANOVA followed by LSD’s post hoc test. *TLR4*, toll-like receptor 4; *MyD88*, myeloid differentiation primary response gene 88; *NF-κB p65*, nuclear factor kappa B p65

### Acupuncture Regulated the Content of Serum IL-1β and IL-10 of CRS Rats

**Fig. 7:**
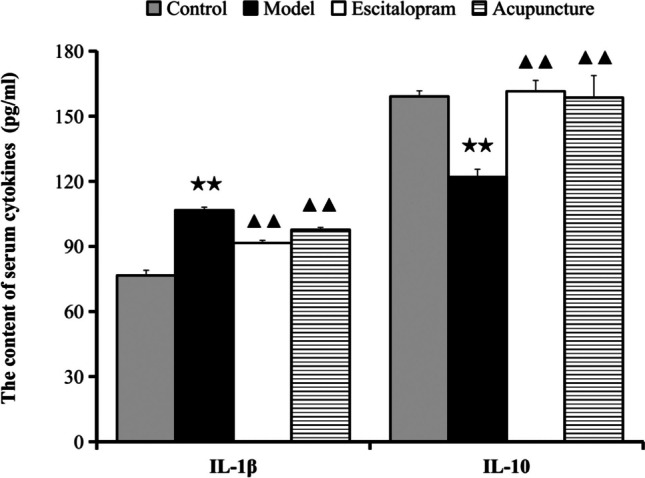
Effect of acupuncture on the content of serum IL-1β and IL-10 of CRS rats. Data were expressed as mean ± S.E. (*n* = 8). IL-1β, *F*=61.750, *P*=0.000; IL-10, *F*=9.698, *P*=0.000. ^★★^*P*˂0.01 compared with the control group; ^▲▲^*P*˂0.05 compared with the model group. One-way ANOVA followed by LSD’s post hoc test. *IL-1β*, interleukin 1 beta; *IL-10*, interleukin 10

As shown in Table 6 and Fig. 7, there were significant differences of serum serum IL-1β and IL-10 among each group (*F*=61.750, *P*=0.000; *F*=9.698, *P*=0.000). Compared with the control group, there was significant expression of increased IL-1β and decreased IL-10 in serum after rats exposed to 21 days of CRS procedures (all *P* < 0.01). Acupuncture intervention obviously reversed the CRS-induced expression of increased IL-1β and decreased IL-10 in serum (all *P* < 0.01).

**Table 6:** Effect of acupuncture on the content of serum IL-1β and IL-10 of CRS rats (*n*=8)

Group	IL-1β (pg/ml)	IL-10 (pg/ml)
Control	76.610±2.432	159.127±2.616
Model	106.687±1.369^★★^	122.005±3.571^★★^
Escitalopram	91.573±1.224^▲▲^	161.509±4.995^▲▲^
Acupuncture	97.663±1.035^▲▲^	158.601±10.148^▲▲^

Notes: Data were expressed as mean ± S.E. (*n* = 8). IL-1β, *F*=61.750, *P*=0.000; IL-10, *F*=9.698, *P*=0.000. ^★★^*P*˂0.01, compared with the control group; ^▲▲^*P*˂0.05, compared with the model group. One-way ANOVA followed by LSD’s post hoc test. *IL-1β*, interleukin 1 beta; *IL-10*, interleukin 10

## Discussion

Currently, merging evidence has confirmed that acupuncture, a complementary and alternative medicine originated from traditional Chinese medicine (TCM), has been accepted worldwide. In this study, we demonstrated some compelling findings on the antidepressant effect of acupuncture through modulating the stress-induced neuroinflammation mediated by TLR4 signaling pathway in a rat model exposed to CRS.

### Acupuncture Exerted Preventive Effect Through Alleviating the CRS-Induced Depression-Like Behaviors

Epidemiological studies have consistently reported that the crosstalk between different stress factors is involved in the pathogenesis of depression [7]. And animal models of depression have provided key insights into investigating the miscellaneous pathogenesis and preventive strategy for depression. Stress-induced depression-like behaviors, including low of interest, anhedonia, or hopelessness, have been generally evaluated to assess the establishment of the animal models of depression [38, 39]. Accumulating studies have identified the involvement of CRS-induced depression-like behaviors in the process of depression [40, 41]. In the present study, the depression rat model was induced by social isolation and CRS to imitate depression-like behaviors evaluated by body weight assessment and sucrose preference test. The findings from the behavioral assessment showed that exposure to continuous social isolation and CRS procedures induced significantly reduced body weight gain and decreased sucrose preference in the model group compared with control group, suggesting the CRS-induced depression-like behaviors. However, the CRS-induced decreased body weight and anhedonia were significantly attenuated after acupuncture intervention, indicating the positively preventive effect of acupuncture. The findings from our current study are consistent with those of previous studies [19, 29, 34].

Accumulating evidence have demonstrated that acupuncture could improve symptoms and quality of life for patients with depression, or alleviate the depression-like behaviors in animal model subjected to depression [32, 33, 30, 42]. Acupuncture has also been suggested to be an adjunct to antidepressants, which may enhance the antidepressant effect and reduce the adverse drug reactions in patients receiving antidepressants [43]. Baihui (GV20) and Yintang (GV29) have been identified to be the main acupoint prescription unit for the relief of nervous system disorders, including depression [43]. In the present study, acupuncture at Baihui (GV20) and Yintang (GV29) was applied to be the acupuncture modulation for the treatment of depression in rats exposed to CRS. The results from the behavioral assessment have indicated that acupuncture at Baihui (GV20) and Yintang (GV29) alleviated the CRS-induced depression-like behaviors. Based on the basic theory of TCM, Baihui (GV 20), located at the vertex where the liver meridian and the governor vessel are convergent, is considered to be involved in regulating the flow of Qi, soothing the liver and activating the mental activity. And Yintang (GV29), located at the forehead and affiliated to the governor vessel, has been verified to calm the mind. Various other studies have also shown that acupuncture at Baihui (GV 20) and Yintang (GV 29) could exert antidepressant and preventive effect by regulating the hippocampal injury and inflammation response induced by stress, which has also been verified in our previous studies [19, 29, 34].

### Acupuncture Reversed the CRS-Induced Neuroinflammation Mediated by TLR4 Signaling Pathway

Presently, accumulating evidence have pointed to the critical role for stress-induced neuroinflammation in the pathogenesis of depression, suggesting that the combined effects of stress on inflammation may synergize to facilitate stress-related depression [44]. Activated microglia has been identified to be a characteristic indicator of the activation of the stress-induced neuroinflammation, which has been reported to be involved in the pathological process of depression [45]. TLR4, a pattern recognition receptor, has been proved to be mainly expressed in microglia and exerts regulatory roles in linking stress and depression [46, 47]. Studies have shown that activation of the TLR4 complex might underlie the pathophysiology of stress-evoked depression [47]. Recent findings have revealed that the TLR4 single gene polymorphisms were associated with suicide and anxiety scores in depression patients, while methylation levels of TLR4-associated CpGs were identified to be associated with the severity of depressive symptoms [48, 49]. Studies have provided evidence that TLR4 signaling was upregulated in newly diagnosed patients with depression [23].

Data from the animal experiment have verified the neuroinflammation-induced depressive-like behavior mediated by the activation of microglia and TLR4 in stress models of depression [28, 50]. Studies have reported that stress increased the expression of ionized calcium binding adaptor molecule 1 (IBA-1) in the hippocampus CA1 region [19], promoted the release of pro-inflammatory cytokines IL-1β, IL-18, IL-6, and TNF-α [19, 51], and reduced the brain derived neurotrophic factor (BDNF) expression in the hippocampus [52]. In the present study, the findings indicated that exposure to CRS procedures obviously activated the hippocampal microglia and elevated the expression of TLR4, MyD88, TRAF6, NF-κB p65, TNF-α, and mRNA expression levels of TLR4 in the hippocampus, increased IL-1β, and decreased IL-10 in serum when compared to that of the control group, suggesting the involvement of the stress-induced activation of neuroinflammation in the pathogenesis of depression. Results from the present study were consistent with the reported studies [28, 51], which predicated the involvement of stress-triggered inflammatory factors and cytokines in the pathological process of depression [53, 50]. However, stress-induced depression-like behaviors were effectively alleviated by inhibiting microglial activation and neuroinflammation via TLR4 pathways [28, 54, 55]. Similarly, the stress-induced activation of neuroinflammation in the present study was significantly alleviated by acupuncture compared to the model group. Acupuncture obviously reversed the increase of TLR4, MyD88, TRAF6, NF-κB p65, TNF-α, and mRNA expression levels of TLR4 in the hippocampus, decreased IL-1β, and elevated IL-10 in serum. Acupuncture exerted potential preventive effect which might be mediated in part by suppressing the neuroinflammation induced by TLR4 signaling pathway.

However, there are shortcomings and limitations of the present study. The present study only preliminarily investigated the effect of acupuncture on ameliorating depression-like behaviors via regulating the neuroinflammation mediated by TLR4 signaling pathway in rats exposed to chronic restraint stress. No specific blocker of TLR4 pathway was used to verify whether acupuncture had specificity in the regulation of TLR4 pathway in the study. Meanwhile, our previous studies have identified the preventive effect of acupuncture in the treatment of depression. Hence, sham acupuncture was not set to be the intervention control in our study. Further research is needed to clarify the precise mechanisms of the antidepressant effect of acupuncture in our future study.

## Conclusion

Conclusively, the present study provided evidence that the stress-induced activation of neuroinflammation mediated by TLR4 signaling pathway might be the key pathway linking stress and depression. Acupuncture exerted potential antidepressant-like effect that might be mediated in part by suppressing the neuroinflammation induced by TLR4 signaling pathway, which may be a promising treatment target to improve current treatments for depression. In the following study of our team, we are continuing to carry out the study of the antidepressant effect of acupuncture based on the present findings.

### Supplementary Information

ESM 1 (MP4 50767 kb)

## Data Availability

The online version contains supplementary material available at the attachment. All data and materials were present the present study.
